# A novel V1a receptor antagonist blocks vasopressin-induced changes in the CNS response to emotional stimuli: an fMRI study

**DOI:** 10.3389/fnsys.2013.00100

**Published:** 2013-12-12

**Authors:** Royce J. Lee, Emil F. Coccaro, Henk Cremers, Rosemary McCarron, Shi-Fang Lu, Michael J. Brownstein, Neal G. Simon

**Affiliations:** ^1^Clinical Neurosciences and Psychopharmacology Research Unit, Department of Psychiatry and Behavioral Neurosciences, The University of ChicagoChicago IL, USA; ^2^Azevan Pharmaceuticals, Inc.Bethlehem, PA, USA; ^3^Department of Biological Sciences, Lehigh UniversityBethlehem, PA, USA

**Keywords:** vasopressin, depression, fMRI, anger, neuropeptides, stress, amygdala, parietal

## Abstract

**Background**: We hypothesized that SRX246, a vasopressin V1a receptor antagonist, blocks the effect of intranasally administered vasopressin on brain processing of angry Ekman faces. An interaction of intranasal and oral drug was predicted in the amygdala.

**Methods**: Twenty-nine healthy male subjects received a baseline fMRI scan while they viewed angry faces and then were randomized to receive oral SRX246 (120 mg PO twice a day) or placebo. After an average of 7 days of treatment, they were given an acute dose of intranasal vasopressin (40 IU) or placebo and underwent a second scan. The primary outcome was BOLD activity in the amygdala in response to angry faces. Secondary analyses were focused on ROIs in a brain regions previously linked to vasopressin signaling.

**Results**: In subjects randomized to oral placebo-intranasal vasopressin, there was a significantly diminished amygdala BOLD response from the baseline to post-drug scan compared with oral placebo-intranasal placebo subjects. RM-ANOVA of the BOLD signal changes in the amygdala revealed a significant oral drug × intranasal drug × session interaction (*F*_(1, 25)_ = 4.353, *p* < 0.05). Follow-up tests showed that antagonism of AVPR1a with SRX246 blocked the effect of intranasal vasopressin on the neural response to angry faces. Secondary analyses revealed that SRX246 treatment was associated with significantly attenuated BOLD responses to angry faces in the right temporoparietal junction, precuneus, anterior cingulate, and putamen. Exploratory analyses revealed that the interactive and main effects of intranasal vasopressin and SRX246 were not seen for happy or neutral faces, but were detected for aversive faces (fear + anger) and at a trend level for fear faces.

**Conclusion**: We found confirmatory evidence that SRX246 has effects on the amygdala that counter the effects of intranasal vasopressin. These effects were strongest for angry faces, but may generalize to other emotions with an aversive quality.

## Introduction

Vasopressin (AVP) is a mediator of social and emotional behavior in many species (Garrison et al., 2012) including humans, and it has been suggested that AVP receptor antagonists might be useful for treating stress-related neuropsychiatric problems including inappropriate aggression, post-traumatic stress disorder, and major depression (Meyer-Lindenberg and Tost, 2012). The hypothesis that these disorders might respond to AVP antagonists is supported by preclinical and clinical studies showing that CNS vasopressinergic signaling is deregulated in patients with these indications (reviewed in Simon et al., 2008).

There are two AVP receptor subtypes in the brain that are potential therapeutic targets: V1a and V1b. Small-molecule V1b receptor antagonists have thus far not proven effective for the treatment of depression (Griebel et al., 2012), which may be due to the relatively low expression and restricted, hypothalamic distribution of the receptor. In contrast, the V1a receptor is the dominant CNS subtype and is found throughout the limbic system and several cortical regions, providing a strong rationale for determining the potential role of this receptor in the regulation of emotion. To this end, SRX246 was developed as a novel, AVPR1a antagonist that penetrates the blood brain barrier and has CNS effects in multiple preclinical models (Ferris et al., 2008; Simon et al., 2008; Fabio et al., 2013). Because there is currently no PET ligand that can be used to establish AVPR1a target engagement, we felt that it was important to demonstrate that SRX246 produced CNS effects in humans after oral dosing before commencing clinical trials in patients. This step is in accord with the growing consensus that evidence of brain penetration and pharmacological effect is a vital early component of drug development for CNS indications (reviewed in Griebel and Holsboer, 2012).

Due to the impermeability of the blood-brain barrier to orally or intravenously administered neuropeptides, human research regarding central vasopressin signaling has relied on intranasal administration of vasopressin. After intranasal administration, a small amount of vasopressin crosses the blood brain barrier (Riekkinen et al., 1987; Born et al., 2002). A series of human studies has found that intranasally administered vasopressin enhances attention to and memory of emotional facial expressions (Thompson et al., 2004, 2006), Several recent studies have examined the effects of intranasal vasopressin on regional brain activity as measured by the fMRI blood oxygen level dependent (BOLD) response to experimental emotional and social stimuli. In total, intranasal vasopressin appears to increase the neural response to socially relevant stimuli in circuits that mediate emotion regulation (subgenual cingulate: Zink et al., 2010 and Brunnlieb et al., 2013b), theory of mind (inferior parietal lobe: Zink et al., 2011; superior temporal sulcus: Brunnlieb et al., 2013a; posterior cingulate, Brunnlieb et al., 2013b), and social recognition (lateral septum; Rilling et al., 2012). Notably, only one of the five studies found evidence for direct vasopressin on amygdala BOLD (Brunnlieb et al., 2013b). The stimuli in this study consisted of line drawings of aversive social interactions, rather than face stimuli. The absence of a direct effect of intranasal vasopressin on amygdala reactivity to faces was initially unexpected (Zink et al., 2010), given the a priori hypothesis that vasopressin would increase amygdala BOLD response to aversive faces. With regards to vasopressin modulation of amygdala response to face stimuli, indirect effects on the amygdala were detected when examining functional connectivity of the subgenual to supragenual cingulate (Zink et al., 2010). Thus, the human vasopressin challenge literature has found that vasopressin has effects on behavior predicted by preclinical models, increasing reactivity to social stimuli. The brain imaging literature has found evidence of altered neural function following vasopressin challenge in a brain regions previously implicated in social cognition. Because vasopressin as a pharmacological probe in these studies lacks specificity for the V1a or V1b receptor, this work has been unable provide specific information regarding which subtype vasopressin receptor is involved. Progress in this area would require either vasopressin V1a or V1b specific agonists safe for human use, or specific antagonists.

A novel small-molecule vasopressin antagonist (SRX246; Azevan Pharmaceuticals) with a high degree of selectivity for the AVPR1a has undergone preclinical and early clinical testing. SRX246 crosses the blood brain barrier and binds to V1a receptors with a high degree of selectivity (Fabio et al., 2013). Its ability to selectively target AVPR1a is reflected in its ability to reverse AVPR1a-mediated stress reactivity and neural response to intruder threat (Ferris et al., 2008). Because it has undergone successful Phase I single-ascending-dose and 14-day multiple ascending dose clinical trials with a benign safety profile (detailed in Fabio et al., 2013), it became available for use as a pharmacological probe of the AVPR1a.

A double-blinded, placebo controlled experiment was conducted using challenge with intranasal vasopressin and treatment with SRX246, an oral AVPR1a antagonist. We asked normal male volunteers to look at emotional faces while their brains were scanned using fMRI. Half of the subjects were then given SRX246 for an average of 7 days, and half were given placebo-containing capsules. An hour before they were re-scanned, half of the subjects in each condition were given intranasal arginine vasopressin (AVP). AVP challenge was used to maximize the chance of finding an effect of AVPR1a blockade in a sample of healthy subjects, who presumably did not exhibit excessive central AVP signaling. We then looked for evidence that brain regions were activated when patients looked at angry faces vs. a fixation point, that vasopressin affected such activation, and that SRX246 blunted the responses seen in the presence or absence of AVP. Because the amygdala is known to express vasopressin AVPR1a receptors (Young et al., 1999; Huber et al., 2005; Stoop, 2012) and to be activated during explicit recognition of emotional (including angry) faces (Derntl et al., 2009), it served as the principal region of interest in the experiment. Secondary analyses examined effects in additional candidate regions found to be modulated by vasopressin in previous research: the temporoparietal junction, precuneus, anterior cingulate, subgenual cingulate, and putamen.

## Methods

### Participants

All study procedures were approved by the Institutional Review Board of The University of Chicago. All subjects provided written, informed consent. Subjects were recruited from the Chicago region with IRB approved advertisements in local media. Because of previous literature indicating the possibility of sexually dimorphic effects of vasopressin (Thompson et al., 2006), only male subjects were studied to preserve statistical power. Twenty-nine healthy male subjects (ages 18–55) were studied after they were verified to meet inclusion/exclusion criteria by medical exam and psychiatric screening with semi-structured SCID and SID-P IV interviews. Inclusion criteria included being medically and psychiatrically healthy, no past history of an Axis I or II psychiatric disorder, no obstruction of either nostril to the olfactory epithelium, normal screening blood and urine tests, non-smoking, normal body weight, right handedness, and no current use of prescription medications or drugs.

### Experimental design

**Figure 1:**
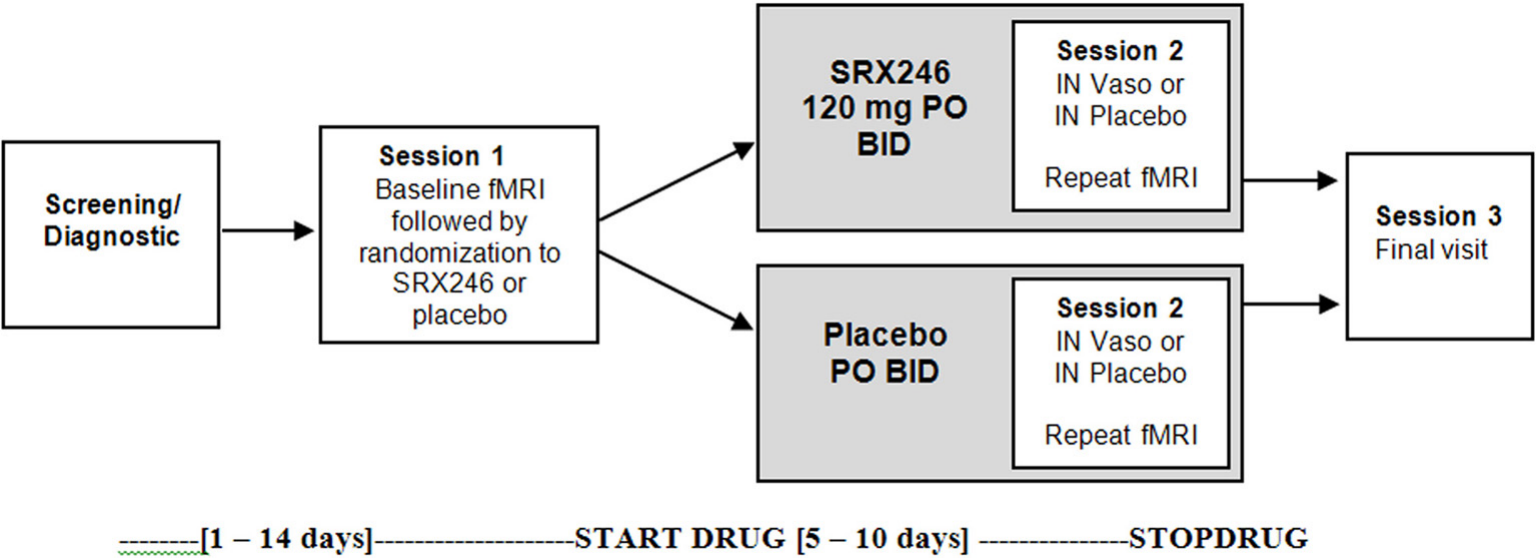
**Study schematic**.

The study was a double-blinded, between subjects design with two fMRI scanning sessions. The first session occurred before any drug administration (Session 1). The second session (Session 2) followed randomization, treatment for a minimum of 5 days with oral SRX246 or placebo, and 45 min after acute administration of intranasal vasopressin or matching placebo (see Figure 1). A between subjects design was chosen to eliminate possible carry over effects of SRX246 or IN vasopressin on the fMRI measures. To mitigate the effect of individual differences in brain reactivity to face stimuli, the design included the first baseline scan to evaluate patterns of change from the first to the second scan.

Two experimental drugs were administered in double-blinded fashion: chronic oral SRX246 and acute intranasal (IN) vasopressin, both with placebo counterparts. After the first scanning session (Session 1, described in the next section), subjects were randomized to receive 5 days of oral SRX246 (120 mg by mouth, twice a day; *n* = 15) or equivalent dosing of the matching pill placebo (*n* = 14). The mean of number of days between the first and second scanning session was 7.3 (*SD* = 1.3); the SRX246 and placebo treatments continued until the day of the second scanning session. The second session began with randomization to either IN AVP or IN placebo. Vasopressin was prepared by the research pharmacy of the University of Chicago General Clinical Research Center. Forty IU synthetic vasopressin (8-arginine-vasopressin, Pitressin, Monarch Pharmaceuticals) was dispensed using Good Clinical Practices into two intranasal atomizers (MAD 300; LMA North America Inc., San Diego CA). IN placebo was prepared from a commercial nasal saline solution to mask the mild scent of Pitressin solution. IN drug was administered in 4 puffs (0.2 mL) per nostril over 15 min by the research staff to subjects reclining on an exam table with their heads tilted back. Subjects rested on the examination table by themselves with the examination room door open until the beginning of the second fMRI scanning session, timed to begin 45 min after IN drug administration. This time point was chosen based on the time course of CSF levels of vasopressin after IN administration (Born et al., 2002) and to remain consistent with previous fMRI studies of IN vasopressin effects.

The first and second scanning sessions were identical. In the scanner, subjects viewed 4 blocks of each emotional facial expression, using stimuli from the Ekman Pictures of Facial Affect stimulus set (angry, neutral, happy, fear). The stimuli were presented over 4 runs. To preserve statistical power, analyses focused on the neural responses to angry faces, based on a priori hypotheses regarding the relevance of angry faces to vasopressin function. Each emotion block lasted 20 s and consisted of 5 faces displayed for 4 s in the center of the screen, with no interstimulus interval. The behavioral task was to identify the valence of the emotion (positive, negative, neutral) by button press. An explicit emotion paradigm was employed based on a large amount of high resolution fMRI data demonstrating that this type of task evokes a readily detectable amygdala response (Pessoa et al., 2002; Habel et al., 2007; Derntl et al., 2009; Dyck et al., 2011). Fixation cross was chosen as a contrast condition rather than neutral faces for this study in order to maximize statistical power by avoiding the variability associated with contrasts of emotional faces with neutral faces. Neutral faces also activate the amygdala (Fitzgerald et al., 2006; Derntl et al., 2009). The extent and variability of this activation reduces BOLD signal intensity when neutral faces are used as a contrast condition (Mattavelli et al., 2013) and reduces the reliability of amygdala response to emotional faces (Johnstone et al., 2005).

fMRI data were acquired using a Philips Achieva Quasar 3T MRI scanner at the Brain Research Imaging Center at The University of Chicago. For identification of landmarks and orientation of follow up scans, low-resolution structural MRI was obtained with a T1-weighted spin-echo sequence (*TR* = 600 ms, *TE* = 10 ms; *FA* = 70°, *FOV* = 23 cm^2^, slice thickness/gap = 4.0/0.5). fMRI images were obtained with high-field functional MRI utilizing T2^*^-weighted echo planar imaging with BOLD (blood oxygenation level dependent) contrast (echo time/*TE* = 20 ms, repetition time/TR of 2000 ms, flip angle of 80°, field of view of 230 mm^2^, 30 4 mm oblique axial slices approximately parallel to the AC-PC line, 0.5 mm slice gap). A modified high efficiency z-shim compensation was applied to the 4 slices covering the orbitofrontal cortex (Du et al., 2007) to minimize susceptibility artifacts. Acceptable signal to noise ratio was confirmed for the ventral brain and medial temporal lobes.

fMRI data were pre-processed using SPM8 software (Wellcome Department of Cognitive Neurology, London). Images were band pass filtered to remove very low frequency drift artifact and high frequency, non-physiologic noise. Images acquired during excessive movement (≥3 mm X, Y, or Z spatial displacement and/or 5° of rotation) were excluded from the analysis. Motion in the three planes was recorded and images were motion corrected relative to the first image of the first run, normalized to a Montreal Neurological Institute template, resampled to 2 mm^3^ voxels, and smoothed with an 8 mm^3^ kernel. T2^*^ functional data of each subject were examined for susceptibility artifacts and/or signal loss near the principal regions of interest.

T-statistical images were generated for the first and second fMRI sessions separately to confirm that the task led to the expected pattern of regional brain activation. To control for Type I error, Family Wise Error (FWE) was utilized for the entire brain region in voxelwide analyses (*p* < 0.05, cluster size > 10 contiguous voxels). Analyses were focused on angry face contrasts given the limited power of the study. An exploratory analysis of other facial emotion conditions is provided in Supplementary Material.

To establish the effect of the IN vasopressin probe in the principal ROI of the left and right amygdala, data were compared between the IN vasopressin and IN placebo group within the sub-sample of subjects randomized to oral placebo (*n* = 14). One-Way ANOVA was conducted on whole-brain contrast images of angry faces vs. fixation point, with the factors of session and IN drug; the hypothesized IN drug effect was tested with the statistical interaction between the two factors by confirming significant clusters of activation within the anatomical amygdala. To balance concerns of Type I and Type II error, correction for multiple comparisons on significant clusters utilized familywise error correction (FWE) within the small volume of the anatomic amygdala as defined by Wake Forest University (WFU) Pickatlas (Maldjian et al., 2003; *p* < 0.05, one-tailed). Parameter estimates (β weights) of average activation were extracted from the anatomical amygdala ROI (WFU Piackatlas) and exported to SPSS 18 (IBM) for statistical analysis. Two separate paired *t*-tests were then conducted in the seven subjects receiving intranasal vasopressin and the seven subjects receiving intranasal placebo. To account for baseline differences, follow up tests were repeated using ANCOVA to compare IN vasopressin vs. IN placebo on the extracted Session 2 amygdala BOLD signal, covarying for Session 1 in the 14 subjects receiving oral placebo.

The primary hypothesis of the study, that SRX246 engages its target by blocking vasopressin effects on the amygdala, was tested with repeated measures (RM) ANOVA for the interaction of the factors of oral drug (SRX246 vs. oral placebo), intranasal drug (IN vasopressin vs. IN placebo), side (left vs. right), and session (Session 1 vs. Session 2) on the extracted parameter estimates of average amygdala BOLD response to angry faces. Repeated Measures (RM) ANOVA was conducted with the between-subjects factors of oral drug and intranasal drug, with the within-subjects factor session. The statistical significance threshold was set at *p* = 0.05, 2-tailed. Significant main effects and/or interactions were followed up with appropriate *post-hoc*, 2-tailed tests. Paired *t*-tests were conducted to compare Session 1 and Session 2 BOLD response in the four drug subgroups. Additionally ANCOVA of Session 2 BOLD response covarying for Session 1, comparing IN vasopressin to IN placebo was conducted in subjects randomized to oral SRX246 and oral placebo.

In exploratory analyses, the effects of SRX246 and vasopressin on BOLD reactivity were assessed in ROIs previously found to be modulated by vasopressin or expressing the AVPR1a: subgenual cingulate (Zink et al., 2010), anterior and posterior cingulate (Zink et al., 2010), precuneus (Brunnlieb et al., 2013a), temporoparietal junction (Rilling et al., 2012; Brunnlieb et al., 2013b), and caudate/putamen (Hammock and Young, 2006). Voxel-wide, whole brain analysis was performed on Session 2 data, comparing the response to angry faces vs. fixation point between the SRX246 to oral placebo treatments. ROI analyses were performed using the corresponding anatomical structure [Automated Anatomical Atlas (AAL) SPM], with the significance threshold set at *p* < 0.05, FWE corrected for the anatomical search volume. Individual differences in the tortuous gyral/sulcal morphology of the temporoparietal region make spatial definitions of it unreliable for group analyses (Tzourio-Mazoyer et al., 2002). Instead, the ROI was a 9 mm sphere centered on MNI coordinates reported in the most recent of a series of studies that have found functional activations in the right temporoparietal junction during theory of mind related tasks (54, −54, 22; Koster-Hale et al., 2013). Average activations within these ROIs at Session 2 were extracted as β weights into SPSS for ANCOVA, covarying for baseline differences. Because this is the first fMRI study of SRX246, exploratory, whole brain exploratory comparisons of SRX246 vs. placebo on BOLD response to angry faces (vs. fixation point) during Session were conducted. Uncorrected results are provided in Table 3.

### Behavioral data

Accuracy and reaction time were recorded and analyzed by RM-ANOVA, with the within subjects factor of session and between subjects factors of oral and intranasal drug. Significant interactions were followed up with *post-hoc t*-tests (2-tailed). To relate the behavioral measures with brain response, change in reaction time and accuracy (Session 2 – Session 1) was correlated with change in amygdala BOLD (Session 2 – Session 1).

### Side effects

The Adverse Events Questionnaire (AEQ) was used to measure a range of possible somatic and psychological side effects. Suicidal symptoms were assessed with the Columbia Suicide Severity Rating Scale (C-SSRS; validation in Posner et al., 2011). Depressive symptoms were measured with the Beck Depression Inventory II (BDI-II; Beck et al., 1996). Differences between drug- and placebo-treated subjects in safety (vital signs and laboratory parameters), side effects (AEQ, BDI-II, CSSR-S), and EKG data (PR interval, QT, QTc) were assessed with a series of separate RM-ANOVAs for each measure.

## Results

### Task-related activations on session 1 and session 2

**Figure 2:**
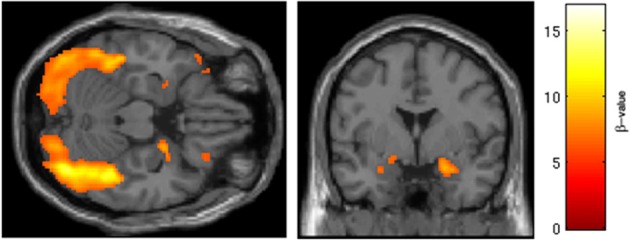
**Regions of BOLD signal intensity for Session 2 angry faces > fixation point**. Results of the Session 2 voxel-wide whole brain analysis for all subjects (*n* = 29), angry faces > fixation point, thresholded at *p* < 0.001 FWE corrected for the entire brain, cluster size > 10 contiguous voxels. A similar pattern of BOLD activation is present albeit with attenuated intensity and size of activated clusters relative to Session 1.

BOLD responses to angry faces vs. fixation point were observed in the visual cortex, right and left amygdala, temporal pole, and ventral prefrontal cortex for Session 1 and 2 (Table 1 for Session 1, Table 2 and Figure 2 for Session 2). Areas of activation were within regions expected to show task-related changes in brain activity. Attenuated intensity and cluster size of BOLD was observed from Session 1 to Session 2 in the entire sample. Contrasts of angry faces vs. neutral faces did not result in measurable amygdala BOLD signal suitable for analysis of drug related effects (see Supplementary Material 3.1^*^).

**Table 1:** **Results of voxel-wide whole brain analysis of Session 1 for contrasts of anger vs. fixation point**.

**Region**	**MNI coordinates**			**Cluster size (voxels)**	***T***	**p_(FWE-corrected)_**
	***x***	***y***	***z***			
Occipital gyrus	40	−76	−14	13,574	18.5	<0.001
Right amygdala	20	−4	−20	763	12.47	<0.001
Superior frontal gyrus	12	66	38	1986	11.66	<0.001
Left middle frontal gyrus	−46	24	50	139	9.73	<0.001
Left amygdala	−18	−6	−14	572	9.5	<0.001
Rectus	−6	54	−18	368	9.34	<0.001
Left temporal pole	−36	26	−28	195	8.31	<0.001
Right temporal pole	42	20	−34	156	7.56	0.002
Left inferior frontal gyrus	−56	34	14	70	7.47	0.002
Left inferior frontal gyrus	−54	40	4	118	7.39	0.002
Left inferior temporal gyrus	−48	10	−38	64	7.05	0.005
Left orbital frontal gyrus	−48	28	−4	62	6.91	0.007
Right inferior frontal gyrus triangular	60	32	2	114	6.8	0.009
Left fusiform	−30	−6	−44	11	6.78	0.009
Supplementary motor area	−4	14	72	23	6.38	0.023

Clusters of significant activation (>10 contiguous voxels) that survive statistical threshold for multiple comparisons (FWE across the entire brain, p < 0.05).

**Table 2:** **Results of voxel-wide whole brain analysis of Session 2 for contrasts of anger vs. fixation point**.

**Region**	**MNI coordinates**			**Cluster size (voxels)**	***T***	**p_(FWE-corrected)_**
	***x***	***y***	***z***			
Occipital gyrus	20	−100	4	13,390	16.87	<0.001
Right inferior frontal gyrus triangular	58	30	16	1150	10.98	<0.001
Right amygdala	22	−4	−16	207	10.03	<0.001
Right hippocampus	24	−30	−4	314	9.18	<0.001
Left inferior orbital frontal	−50	22	−16	1353	8.82	<0.001
Right inferior orbital frontal	42	34	−14	140	8.38	<0.001
Left hippocampus	−20	−34	−2	161	8.12	0.001
Right fusiform	30	−6	−44	52	7.26	0.003
Left cerebellum	−16	−78	−40	84	7.22	0.004
Right cerebellum	28	−76	−40	26	7.01	0.005
Right temporal pole	46	20	−30	68	6.93	0.007
Left temporal pole	−26	18	−30	23	6.89	0.007
Left amygdala	−28	0	−20	11	6.85	0.008
Left amygdala	−18	0	−10	48	6.70	0.011

Clusters of significant activation (>10 contiguous voxels) that survive statistical threshold for multiple comparisons (FWE across the entire brain, p < 0.05).

### Effect of in vasopressin

**Figure 3:**
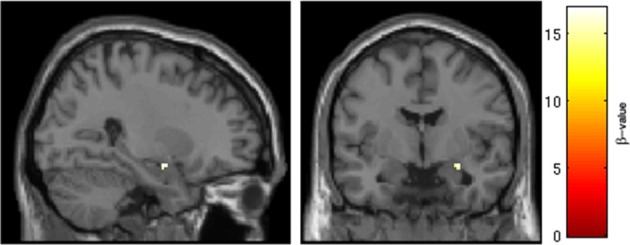
**Intranasal drug × session interaction in the region of the right amygdala**. Results of ROI analysis of the amygdala with One-Way ANOVA in subjects randomized to oral placebo (*n* = 14). A cluster in the right amygdala survived statistical thresholding (*p* < 0.05, FWE corrected for the small volume of the anatomical amygdala). Pictured are voxels surviving the statistical threshold an interaction of session (Session 1 > Session 2) × drug (IN placebo > IN vasopressin). Follow-up testing revealed that SRX246 blocked the effect of IN vasopressin in the right amygdala.

One-Way ANOVA in the subsample of 14 subjects who did not take SRX246 revealed a significant interaction of session × IN drug within both the left and right amygdala (*p* < 0.05, FWE corrected for the volume of the region; depicted in Figure 3). Follow-up paired *t*-tests comparing amygdala BOLD intensity across the first and second session revealed that in subjects receiving IN vasopressin, BOLD signal intensity significantly decreased in the right amygdala [*t*_(1, 6)_ = −2.560, *p* < 0.05] and at a trend level in the average of both sides [*t*_(1, 6)_ = −2.058, *p* = 0.09], but no effect was seen in the left amygdala. No significant pairwise difference in amygdala BOLD between the two sessions was seen in the subjects receiving IN placebo. ANCOVA of the amygdala BOLD response in subjects receiving oral placebo produced similar results, with IN vasopressin associated with significantly decreased BOLD response at Session 2, controlling for Session 1 BOLD response, in the right amygdala [*F*_(1, 13)_ = 5.013, *p* < 0.05] and at a trend level for the combined left and right amygdala [*F*_(1, 13)_ = 4.538, *p* = 0.057]. No effect was found in the left amygdala. See Supplementary Material 3.2^*^ for analyses of other emotion conditions.

### Primary hypothesis: interaction of oral SRX246 and intranasal vasopressin on amygdala bold response

**Figure 4:**
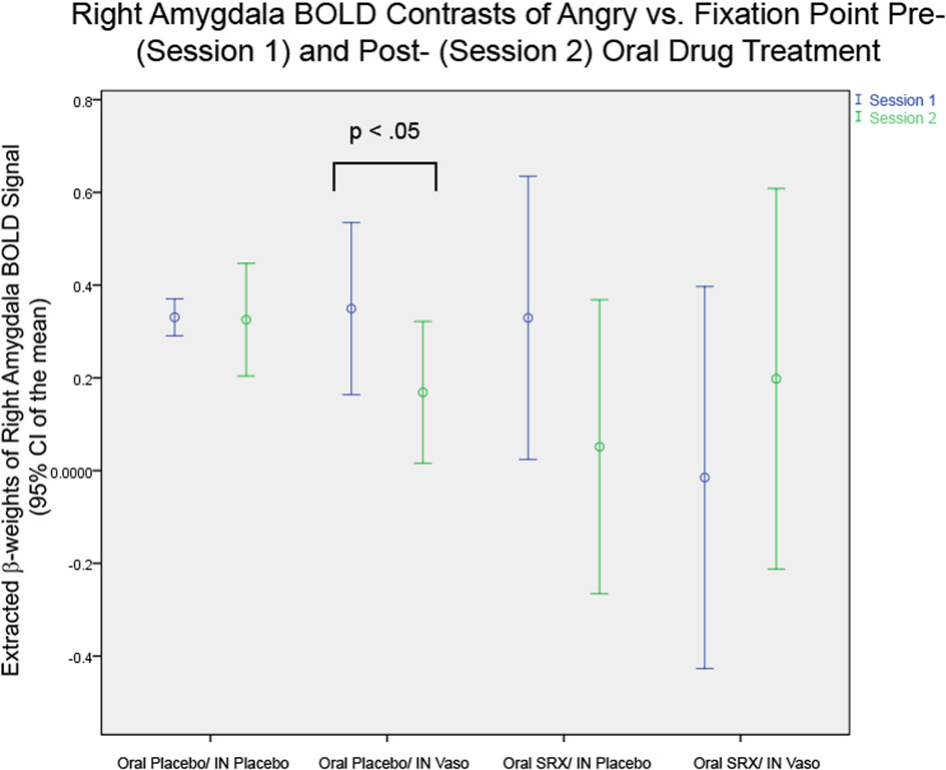
**Right Amygdala BOLD in the Four Drug Subgroups: Significant differences are seen in paired *t*-tests between Session 1 and 2 only in the subgroup of subjects randomized to oral placebo and intranasal vasopressin (*p* < 0.05)**.

The primary hypothesis that SRX246 blocks effects of vasopressin on the amygdala was confirmed. RM ANOVA of extracted parameter estimates from the left and right amygdala in the entire sample revealed a significant 3 way interaction of session × oral drug × IN drug in contrasts of angry faces vs. fixation point [*F*_(1, 25)_ = 4.353, *p* < 0.05]. Follow up testing of the interaction in the four drug subgroups with paired *t*-tests comparing Session 1 to Session 2 BOLD response in the left and right amygdala confirmed that a significant Session 1 vs. Session 2 difference was found only in the subgroup of subjects randomized to oral placebo and IN vasopressin (as described in the Effect of IN Vasopressin). Subjects randomized to oral SRX246 and IN vasopressin did not show a Session 1 to Session 2 difference [*t*_(1, 7)_ = 0.819, *p* = 0.44 (Figure 4)]. ANCOVA of Session 2 amygdala BOLD, covarying for Session 1, confirmed that in subjects taking oral SRX246, no effect of IN vasopressin was seen in the combined amygdala [*F*_(1, 14)_ = 0.384, *p* = 0.55], right amygdala [*F*_(1, 14)_ = 0.067, *p* = 0.8], or left amygdala [*F*_(1, 14)_ = 1.063, *p* = 0.32]. There was no main effect of SRX or vasopressin on amygdala BOLD response. See Supplementary Material 3.3^*^ for analyses of other emotion conditions.

### Secondary analyses

Cross-sectional, Session 2 comparison of SRX246 (*n* = 15) vs. oral placebo (*n* = 14) revealed clusters of activation that survived small volume correction in contrasts of angry faces vs. fixation point within the ROIs of the right sided temporoparietal junction, precuneus, anterior cingulate gyrus, and putamen.

**Figure 5:**
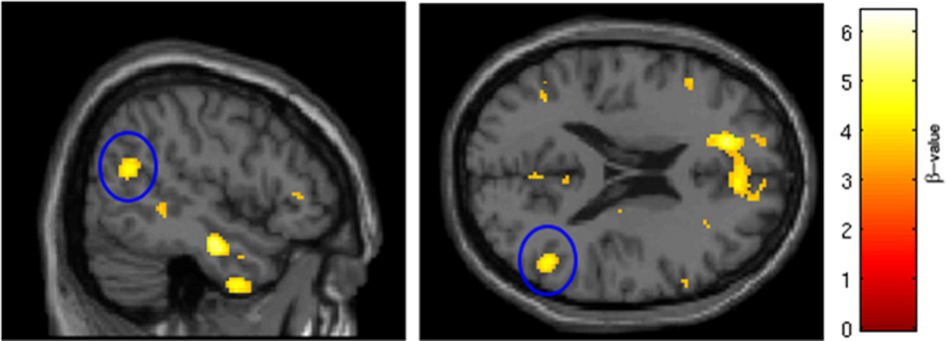
**SRX246 associated blunting of right TPJ to angry faces**. The cluster (circled in blue) of significantly blunted BOLD response within the right temporoparietal junction in the subjects randomized to SRX246 vs. placebo, angry faces > fixation point (*p* < 0.005, FWE corrected for the small volume of the function right TPJ, cluster size = 107 voxels; MNI coordinates = 50, −58, 20).

SRX246 compared with oral placebo was associated with significantly diminished BOLD signal intensity in the right temporoparietal junction [*p* < 0.005, FWE corrected for the functional ROI in the right temporoparietal junction; *t*_(1, 27)_ = 4.52; cluster size = 107 voxels; 50, −58, 20; Figure 5]. ANCOVA confirmed that the difference remained significant after controlling for Session 1 BOLD intensity [*F*_(1, 26)_ = 6.478, *p* < 0.05].

**Figure 6:**
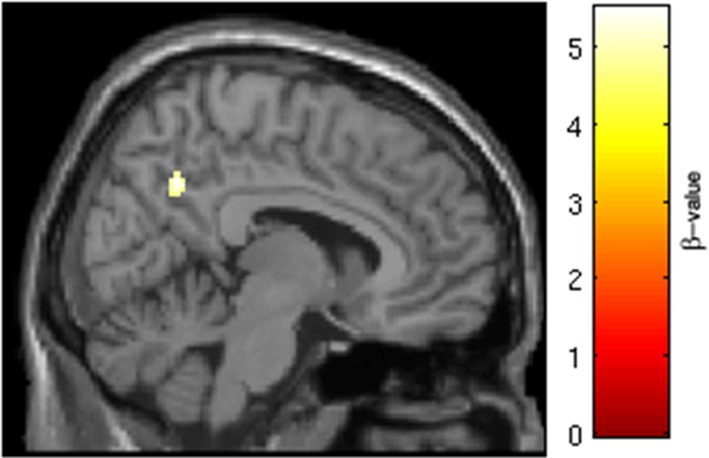
**SRX246 associated blunting of right precuneus to angry faces**. Cluster of significantly blunted BOLD response within the right precuneus in the subjects randomized to SRX246 vs. placebo, angry faces > fixation point (*p* < 0.05, FWE corrected for the small volume of the anatomical precuneus, cluster size = 33 voxels; MNI coordinates = 8, −56, 38).

SRX246 was associated with significantly diminished BOLD activity in a cluster within the right precuneus (*p* < 0.05, FWE corrected for the small volume of the anatomical precuneus, cluster size = 33 voxels; MNI coordinates = 8, −56, 38; Figure 6). ANCOVA revealed that the difference remained significant after controlling for Session 1 BOLD intensity [*F*_(1, 26)_ = 6.208, *p* = 0.02].

**Figure 7:**
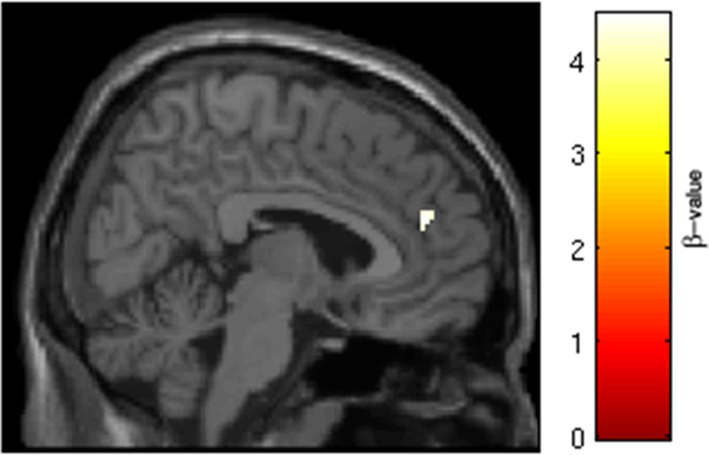
**SRX246 associated blunting of right anterior cingulate to angry faces**. SRX246 was associated with reduced BOLD response in a cluster of the right anterior cingulate [*p* < 0.05 FWE for small volume of the right anterior cingulate. *t*_(1, 27)_ = 4.34, cluster size = 20 voxels; MNI coordinates = 6, 46, 22].

SRX246 was associated with reduced BOLD response in a cluster of the right anterior cingulate [*p* < 0.05 FWE for small volume of the right anterior cingulate, *t*_(1, 27)_ = 4.34, cluster size = 20 voxels; MNI coordinates = 6, 46, 22; Figure 7]. ANCOVA revealed that the difference remained significant after controlling for Session1 BOLD intensity [*F*_(1, 26)_ = 14.473, *p* = 0.001].

**Figure 8:**
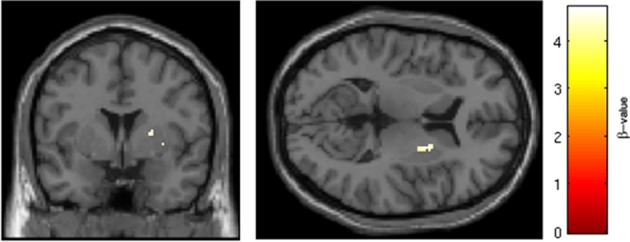
**SRX246 associated blunting of right putamen to angry faces**. SRX246 was associated with reduced BOLD response in two clusters within the right putamen [*p* < 0.05, FWE corrected for small volume, *T*_(1, 27)_ = 4.7 and 4.55, cluster size = 18 and 17 voxels; MNI coordinates = 22, 2, 8 and 28, 12, −8, respectively].

SRX246 was associated with reduced BOLD response in two clusters within the right putamen [*p* < 0.05, FWE corrected for small volume, *T*_(1, 27)_ = 4.7 and 4.55, cluster size = 18 and 17 voxels; MNI coordinates = 22, 2, 8 and 28, 12, −8, respectively; Figure 8]. ANCOVA revealed the difference remained significant after controlling for Session 1 BOLD intensity [*F*_(1, 26)_ = 19.724, *p* < 0.001].

Clusters of significant activation from exploratory voxel-wide whole brain analysis are presented in Table 3, at the uncorrected statistical threshold of *p* < 0.001, one-tailed. No main effects of IN vasopressin were detected in the above ROIs. See Supplementary Material 3.4^*^ for exploratory analyses of other emotion conditions.

**Table 3:** **Exploratory findings of regional BOLD signal blunting with SRX246**.

**Region**	***x***	***y***	***z***	**Cluster size (voxels)**	***T***	**p_(uncorrected)_**
Anterior cingulate/DMPFC	12	40	44	1773	6.41	<0.001
Right inferior temporal	48	−14	−22	227	6.21	<0.001
Right precuneus	8	−56	38	243	5.50	<0.001
Right putamen	18	−2	10	169	5.27	<0.001
Right inferior temporal	44	0	−44	287	5.18	<0.001
Right hippocampus	32	−18	−10	100	5.14	<0.001
Cerebellum	26	−50	−42	290	5.13	<0.001
Cerebellum	−28	−42	−42	56	4.76	<0.001
Right putamen	28	12	−8	157	4.55	<0.001
Right superior temporal	50	−58	20	108	4.52	<0.001
Cerebellum	46	−54	−44	13	4.19	<0.001
Right inferior frontal triangular	48	34	4	32	4.01	<0.001
Left putamen	−18	6	14	12	3.99	<0.001
Left precuneus	−10	−56	46	50	3.98	<0.001
Cerebellum	−28	−82	−38	33	3.97	<0.001
Anterior cingulate	−4	18	24	10	3.96	<0.001
Left middle temporal	−60	−26	−16	23	3.96	<0.001
Right inferior frontal	60	18	16	29	3.95	<0.001
Right fusiform	28	−32	−18	17	3.95	<0.001
Right middle temporal	46	−36	0	62	3.91	<0.001
Cerebellum	18	−48	−24	59	3.90	<0.001
Left inferior frontal triangular	−46	20	18	37	3.88	<0.001
Cerebellum	−28	−56	−18	45	3.87	<0.001
Cerebellum	−18	−66	−42	11	3.80	<0.001
Cerebellum	−10	−62	−32	31	3.71	<0.001
Cerebellum	−22	−68	−20	20	3.59	0.001

Results of the exploratory whole-brain comparison of SRX246 vs. placebo. Listed are clusters of significant activation (>10 contiguous voxels) within the corresponding brain structures that survive statistical threshold (p < 0.001, uncorrected for multiple comparisons).

### Behavioral effects

For accuracy, RM-ANOVA revealed no main effects or interactions for session, IN drug, or oral drug. For reaction time, RM-ANOVA revealed a significant effect of time [*F*_(1, 24)_ = 15.05, *p* < 0.05], with reaction time to Angry faces decreasing from Session 1 (423.41 ms, *SD* = 130.937) to Session 2 (342.414 ms, *SD* = 158.743). A trend level interaction was detected for session × oral drug [*F*_(1, 24)_ = 3.435, *p* = 0.08]. Follow up testing with paired *t*-tests comparing Session 1 to Session 2 revealed that subjects randomized to oral placebo showed a significant decrease in reaction time to angry faces from Session 1 to Session 2 [*t*_(1, 13)_ = 4.773, *p* < 0.001]; subjects randomized to SRX246 also showed a decrease but the difference was not significant [*t*_(1, 13)_ = 1.182, *p* = 0.26].

Change in amygdala BOLD response to angry faces (Session 2 – Session 1) was negatively correlated with the change in number of hits (# correct valence identifications for Session 2 – Session 1). This was true for the average of left and right amygdala together (*r* = −0.520, *p* = 0.005, *n* = 28), for the left amygdala (*r* = −0.462, *p* = 0.013) and the right amygdala (*r* = −0.527, *p* = 0.004). Thus, the decreased BOLD response from Session 1 to Session 2 seemed to predict improved performance. There was no relationship between change in amygdala BOLD response to angry faces and reaction time measures.

### Safety and side effects

There were no serious or unexpected adverse events. SRX246 was not associated with change in AEQ subscores, BDI-II, vital sign parameters, laboratory parameters, CSSR-S score, or urine specific gravity.

## Discussion

Chronic treatment with SRX246, a novel AVPR1a antagonist, blunts the effect of acute vasopressin administration on the functional response of the amygdala to angry faces in healthy males. An effect of intranasal vasopressin on subcortical processing of emotional facial expressions was confirmed: in healthy males, vasopressin enhanced accommodation, as reflected in a decrease from Session 1 to Session 2, of the amygdala to angry faces. This effect was effectively blocked by pretreatment with SRX246. Additional main effects of SRX246 were found on cortical processing of angry faces in the right side of the brain within the ROIs of the temporoparietal junction, precuneus, anterior cingulate, and putamen. These novel findings provide the first evidence for AVPR1a signaling in a neural circuit that mediates processing of social and emotional information (Saxe and Kanwisher, 2003; Zink and Meyer-Lindenberg, 2012).

The findings add to a growing literature regarding the role of vasopressinergic AVPR1a signaling in human social and emotional behavior. Genetic variation in the promoter region of AVPR1a has been associated with risk for autism, in which social deficits are the core symptom (Kim et al., 2002; Wassink et al., 2004). The same genetic polymorphisms have been linked to an altered functional response of the amygdala to fearful and angry faces in healthy adults (Meyer-Lindenberg et al., 2009).

Preclinical studies have demonstrated that vasopressin plays an important role in social recognition. We observed that IN vasopressin was associated with accommodation of the BOLD response of the amygdala to angry faces and that SRX246 pre-treatment blocked this effect. These results are consistent with a role for vasopressin in social recognition in humans and provide the first evidence for the involvement of AVPR1a in this process. The direction of the effect of IN vasopressin, specifically decreased BOLD signal, differed with a previous animal model finding suggesting excitation as an expected outcome (Huber et al., 2005). Two potential explanations for this discrepancy can be put forward. One is found in rodent models, where AVPR1a signaling in the lateral septum is a necessary and sufficient condition for social recognition (Allaman-Exertier et al., 2007). Interestingly, the lateral septum tends to inhibit the amygdala, an action which likely facilitates social approach behaviors (Thomas et al., 2012). Whether the lateral septum, or its functional human equivalent, facilitates social recognition by suppression of the amygdala in humans is unknown. The second is that amygdala habituation to repeated presentation of emotional faces has been established in humans (Hariri et al., 2002). Our data indicate that vasopressin signaling through the AVPR1a plays a role in this process and that AVPR1a antagonism can modulate this effect.

Secondary analyses of the main effects of SRX246 showed that treatment with the AVPR1a antagonist reduced the response to angry faces in the right sided temporoparietal junction, precuneus, anterior cingulate, and putamen. The inhibitory effect of AVPR1a blockade on temporoparietal junction activation to angry faces is consistent with previous findings of vasopressinergic modulation of this brain region during processing of social and emotional information (Zink et al., 2011; Rilling et al., 2012; Brunnlieb et al., 2013b). The TPJ is involved in cognitive processes such as theory of mind and psychological perspective taking (Saxe and Kanwisher, 2003; Decety and Grezes, 2006) that play a fundamental role in human social interactive behavior. Hyperactivity has been noted in this region in anxious patients during negative social interactions (McClure-Tone et al., 2011) and altered function is considered a potential mechanism in disorders such as autism (Kana et al., 2012). Given that the TPJ is responsive to the emotional, social, and moral aspects (Kret et al., 2011; Koster-Hale et al., 2013) of stimuli involving social interaction, stress-related over-mentalizing during negative social interactions, mediated in part through the TPJ, may be a psychopathological mechanism amenable to treatment with AVPR1a antagonists. Our results raise the possibility that the TPJ may represent a novel treatment target in stress related disorders, although an important question in this context is whether the effects of SRX246 are mediated directly by AVPR1a in the region itself or indirectly via connected substructures such as the amygdala, lateral septum, posterior cingulate, or thalamus that, based on studies in rodents and non-human primates, are known to express vasopressin receptors (Young et al., 1999; Phelps and Young, 2003).

AVPR1a modulation of the precuneus is consistent with previous findings that IN vasopressin increased precuneus activity during a simulated aggressive social interaction (Brunnlieb et al., 2013a). Precuneus activity has been reported in fMRI studies of face processing (metaanalysis in Fusar-Poli et al., 2009), and has specifically been associated with social recognition (Lee et al., 2013). In general, the precuneus is thought to play a role in self-awareness and higher cognitive processes above and beyond sensory discrimination (reviewed in Cavanna and Trimble, 2006). The clinical potential of pharmacological modulation of the precuneus is suggested by a link to biological risk factors for anxiety and depression (Rogers et al., 2013).

That the AVPR1a antagonist treatment resulted in blunting of the anterior cingulate response to angry faces is consistent with previous research. IN vasopressin increases anterior cingulate activity during processing of social interactions (Brunnlieb et al., 2013a) and prevents supragenual cingulate deactivation during viewing of angry and fearful faces (Zink et al., 2010). The anterior cingulate is activated by viewing of emotional faces (Fusar-Poli et al., 2009). Like the precuneus, it is also implicated in social cognition (reviewed in Frith and Frith, 2003). Increased reactivity of the anterior cingulate to negatively valenced stimuli is a consistent finding in major depressive disorder (reviewed in Hamilton et al., 2013); thus modulation of the anterior cingulate via AVPR1a antagonism may well have clinical utility. The putamen expresses vasopressin receptors (Hammock and Young, 2006) and is activated by viewing of angry faces (Strauss et al., 2005). Our finding that SRX246 blunted the response in this region is in accord with these findings and indicates a prominent role for the V1a receptor subtype. A recent review of fMRI studies of face processing in major depression has found a pattern of putamen hyperreactivity to angry faces (Stuhrmann et al., 2011). Future studies should investigate the possibility that blocking AVPR1a in depressed patients reverses putamen hyperreactivity to aversive stimuli.

Given the design of the study and results from the exploratory analyses on the processing of emotions other than anger, the effects of vasopressin modulation and SRX246 may extend beyond only angry faces to the processing of aversive emotional expressions more generally. Such an interpretation would be consistent with the findings of Thompson and others (2006), who found that intranasal vasopressin increased tone of the corrugator supercilii muscle, a facial expression associated with response to threat common to anger and fear. This possibility is interesting in terms of potential treatments for stress-related disorders, but given the limitations of the current study, additional work is needed to fully characterize the effects of vasopressin and AVPR1a receptor antagonism on specific emotional responses.

Our study presents the first translational investigation of a novel, first-in-class AVPR1a antagonist in a vasopressin challenge, emotional processing paradigm. Some limitations of the study are worth mentioning regarding the interpretation of the results. To optimize reliability, the sample was made as homogenous as possible in terms of age range, gender, and psychiatric profile. To preserve statistical power for the primary comparisons, the analysis focused on the amygdala and contrasts involving angry facial expressions. For feasibility reasons, the study was not powered to detect emotion specific effects of SRX246. Secondary analyses regarding main effects of SRX246 were similarly restricted to a subset of brain regions previously identified to be affected by vasopressin signaling. The limited power of the study makes it likely that significant effects of vasopressin and SRX246 on other brain regions were not detected. Finally, interpretation of the fMRI BOLD signal as responsive to pharmacological manipulations requires the inference that the drug has a direct effect on regional brain activity. While the results, when combined with extensive *in vitro* studies that demonstrate exceptional selectivity and selectivity for the V1a receptor and *in vivo* results in preclinical models (e.g., Ferris et al., 2008; Fabio et al., 2013) strongly suggest target engagement, definitive evidence requires studies with a PET or SPECT ligand. Unfortunately, no such ligands are available.

In conclusion, the results provide the initial demonstration in humans that blockade of AVPR1a with SRX246 significantly reduces the effect of intranasally administered vasopressin on the response of the amygdala to angry face stimuli as measured by the fMRI BOLD response. Additional effects of SRX246 were observed on responses in the temporoparietal junction, putamen, precuneus, and anterior cingulate. These findings extend a growing body of evidence establishing the importance of vasopressin signaling in the processing of social and emotional stimuli. Because exaggerated responses to negatively valenced emotional stimuli in circuits that include these structures are characteristic of several stress-related psychiatric disorders, the ability of SRX246, a novel AVPR1a antagonist, to attenuate the response to angry faces supports the potential of AVPR1a antagonism as a new approach to the treatment of these indications.

## Author contributions

Royce J. Lee conceptualized the experiment, designed the study, devised the intranasal approach, and wrote the manuscript. Emil F. Coccaro provided advisory support, helped to design the experiment, and contributed to the manuscript. Henk Cremers played a key role in processing of fMRI data, choice of analysis parameters, and assistance with critical revisions. Rosemary McCarron designed the fMRI experiment and played a key role in fMRI data analysis. Shi-Fang Lu contributed to the design and the study. Michael J. Brownstein helped to design the experiment and contributed to the manuscript. Neal G. Simon helped to design the experiment and contributed to the manuscript.

## Conflict of interest statement

Emil F. Coccaro is on the scientific advisory board for Azevan. Shi-Fang Lu, Michael J. Brownstein, and Neal G. Simon hold equity in Azevan Pharmaceuticals. The authors declare that the research was conducted in the absence of any commercial or financial relationships that could be construed as a potential conflict of interest.
