# Supplementary material for: A novel V1a receptor antagonist blocks vasopressin-induced changes in the CNS response to emotional stimuli: an fMRI study
Source: Front Syst Neurosci. 2013 Dec 12;7:100. doi: 10.3389/fnsys.2013.00100 (PMC3859978; doi:10.3389/fnsys.2013.00100)
Supplement: Supplementary file 1 [file DataSheet1.PDF]

3.1*: Task Related Activations: As predicted, the neutral face condition represented a problematic contrast for angry faces in the population studied. Extracted parameter estimates of left and right amygdala BOLD contrast to angry vs. neutral faces consistently resulted in very low mean parameter estimates with a high degree of variability (see **Table 1** below).

	N	Mean	Std. Deviation
Session 1 L Angry vs. Fix	29	.2808	.32376
Session 1 L Angry vs. Neutral	29	-.0338	.40865
Session 2 L Angry vs. Fix	29	.2044	.32739
Session 2 L Angry vs. Neutral	29	.0131	.54527
Session 1 R Angry vs. Fix	29	.2395	.34450
Session 1 R Angry vs. Neutral	29	-.0827	.40118
Session 2 R Angry vs. Fix	29	.1864	.32320
Session 2 R Angry vs. Neutral	29	-.0052	.64982

Table 1: Means and Standard Deviations of Extracted Parameter Estimates of Average Left and Right Amygdala BOLD Signal Contrast: Average signal within the anatomically defined ROI of the amygdala (WFU atlas) confirmed that the neutral condition was not a suitable contrast condition for this repeated measures study. Mean BOLD parameter estimates were close to zero with a high degree of variability.

3.2*: Effects of Intranasal (IN) Vasopressin on Processing of Facial Emotions Other than Anger: In exploratory analyses we examined the effects of IN vasopressin on amygdala response to emotional faces other than angry ones. These included happy, fearful, and neutral faces vs. fixation cross, in addition to an all faces condition and an average of anger and fear faces (aversive faces). The tables below summarize the results of comparisons in the 14 subjects randomized to oral placebo. **Table 2** below provides the results of 2-tailed, independent samples t-tests of Session 2 data comparing extracted amygdala BOLD signal intensity in subjects randomized to IN vasopressin versus IN placebo. Where the significance values were at a trend level or p < .05, IN vasopressin was associated with lower amygdala BOLD signal intensity.

	**Fear**	**Happy**	**Neutral**	**All Faces**	**Anger + Fear**
**Left Amygdala**	t (1, 12) = 1.492<br>p = .16	t (1, 12) = .996<br>p = .34	t (1, 12) = .484<br>p = .65	t (1, 12) = 1.81<br>p = .10	t (1, 12) = 2.393<br>p = .03
**Right Amygdala**	t (1, 12) = 1.624<br>p = .13	t (1, 12) = 1.32<br>p = .211	t (1, 12) = .787<br>p = .45	t (1, 12) = .2.206<br>p = .05	t (1, 12) = 2.483<br>p = .03
**Bilateral Amygdala**	t (1, 12) = .1.788<br>p = .09	t (1, 12) = 1.172<br>p = .26	t (1, 12) = .671<br>p = .52	t (1, 12) = 2.042<br>p = .06	t (1, 12) = 2.092<br>p = .06

Table 2: Independent Samples t-tests Comparing the Effects of IN Vasopressin to IN Placebo in Subjects Randomized to Oral Placebo on Amygdala Average BOLD Signal Intensity in Emotions Other Than Anger Contrasted with Fixation Cross: The results of t-tests comparing average BOLD signal intensity from the anatomical amygdala (WFU Atlas) in contrasts of facial emotion conditions other than anger vs. fixation cross. p-values are 2-tailed

and uncorrected for multiple measures. Data is presented on subjects randomized to oral placebo (n = 14) to remove the potentially confounding effects of SRX246.

To look for a change in amygdala BOLD signal from Session 1 to Session 2 in response to faces other than angry ones, paired t-tests were run in subjects randomized to oral placebo, removing the potentially confounding effect of oral SRX246. There were no significant changes. In subjects randomized to IN vasopressin, trend level decreases from Session 1 to Session 2 were found in the right amygdala and combined left and right amygdala for the combination of Anger and Fear faces. **Table 3** below reports on the results of paired t-tests comparing Session 1 and Session 2 in the seven subjects randomized to oral placebo and IN vasopressin.

	**Fear**	**Happy**	**Neutral**	**All Faces**	**Anger + Fear**
**Left Amygdala**	t (1, 6) =1.868<br>p = .11	t (1, 6) = .741<br>p = .49	t (1, 6) = .147<br>p = .65	t (1, 6) = 1.580<br>p = .17	t (1, 6) = 1.925<br>p = .10
**Right Amygdala**	t (1, 6) = 1.270<br>p = .25	t (1, 6) = .221<br>p = ..83	t (1, 6) = 1.181<br>p = .45	t (1, 6) = 1.656<br>p = .15	t (1, 6) = 2.145<br>p = .08
**Bilateral Amygdala**	t (1, 6) = 1.645<br>p = .15	t (1, 6) = .528<br>p = .62	t (1, 6) = .379<br>p = .72	t (1, 6) = 1.664<br>p = .15	t (1, 6) = 2.076<br>p = .08

Table 3: Results of Paired t-tests Comparing Session 1 and Session 2 in Subjects Randomized to IN Vasopressin (N = 7): In the subjects randomized to oral placebo and IN vasopressin, IN vasopressin was associated with trend level decreases from Session 1 to Session 2 in bilateral and right sided amygdala BOLD signal intensity (2-tailed, uncorrected).

Summary: The results of the exploratory analyses of vasopressin effects on amygdala BOLD response to emotional faces suggest that the effects of vasopressin may not be exclusive to the processing of anger. A cross sectional effect is seen for vasopressin on all faces in the right amygdala and at a trend level, in the bilateral amygdala for fear faces. Significant vasopressin effects were also detected when examining aversive faces. Trend-level longitudinal effects were seen in the right amygdala and combined amygdala in the seven subjects randomized to IN vasopressin and oral placebo, while no such trend was seen in any of the other three drug conditions. The overall pattern of results suggests that amygdala processing of the angry face condition, and perhaps aversive facial expressions in general, may be more sensitive to vasopressin modulation. Caution is warranted in drawing strong inferences. This study was not powered for the detection of emotion specific effects. It is possible that vasopressin has effects on amygdala processing of facial expressions other than anger that were simply not detected in this study.

3.3*: Exploratory Analyses of the Interaction between Oral SRX246 and IN Vasopressin on Amygdala BOLD Response to Happy, Fear, and Neutral Faces: These analyses were conducted in parallel fashion with those conducted testing the primary hypothesis of a specific effect on processing of angry faces. Given the exploratory nature of these analyses, the data reported are uncorrected for multiple measures.

For fear vs. fixation, RM-ANOVA detected no significant main effects or interactions. Trend level interactions were found for session x side x oral drug x intranasal drug (F (1, 25) = 3.532, p = .07) and session x oral drug x intranasal drug (F (1, 25) = 3.178, p = .09).

For neutral vs. fixation, RM-ANOVA revealed no significant interaction of session x oral drug x IN drug (F (1, 25) = 1.55, p = .244) or side x session x oral drug x IN drug (F (1, 25) = .164, p = .69). A significant interaction between side x session x IN drug was detected (F (1, 25) = 4.725, p = .04). However, follow-up paired t-tests in both right and left amygdala in the two subsamples randomized to either IN vasopressin or IN placebo revealed no significant differences between Session 1 and Session 2. Right amygdala BOLD decreased nonsignificantly in subjects randomized to IN vasopressin (t (1, 14) = 1.638; p = .124).

For happy vs. fixation, RM-ANOVA revealed no significant interaction of Session x Oral Drug x Intranasal Drug (F (1, 26) = .208, p = .65) or interaction of Side x Session x Oral x IN (F (1, 26) = .022; p = .88).

Examining aversive faces as a group (average of anger + fear vs. fixation), a significant interaction was found for session x oral drug x IN drug (F (1, 25) = 5.360; p = .029). The interaction was followed up by separately examining subjects randomized to oral placebo and oral SRX246. In subjects randomized to oral placebo, independent samples t-tests revealed that IN vasopressin versus IN placebo was associated with decreased amygdala BOLD (t (1, 12) = 2.393, p = .034). On the other hand, no difference between IN vasopressin and IN placebo was found in subjects randomized to SRX246 (t (1, 13 = -.364, p = .722). ANCOVA of Session 2 data, covarying for Session 1, revealed that in subjects randomized to oral placebo, IN vasopressin was still associated with decreased combined (left and right) amygdala BOLD signal intensity relative to IN placebo (F (1, 11) = 5.693, p = .036). In subjects randomized to oral SRX246, there was no difference between IN vasopressin and IN placebo (F (1, 12) = .042, p = .840). Examining change from Session 1 to Session 2, paired t-tests revealed a trend decrease in subjects randomized to oral placebo and IN vasopressin (n = 7; t (1, 6)=-2.07, p = .083). No such effect or trend was seen in the three other drug conditions.

For all faces vs. fixation, a trend level effect was seen for the interaction of session x oral drug x IN drug (F (1, 25) = 3.178, p = .09). A trend level effect was also seen for side x session x oral drug x IN drug (F (1, 25) = 3.532, p = .07).

Summary: Exploration of the interaction of SRX246 and IN vasopressin on amygdala processing of emotional facial expressions other than anger provided some evidence that the interactive effects of SRX246 and IN vasopressin are most readily seen in angry faces. Trend level interactions between session, SRX246, and vasopressin were detected for fear faces. Significant effects were found for aversive faces, suggesting that the interactive effects are not confined to angry faces alone, but may rather be to aversive faces in general. However, a larger study with greater power is needed in order to adequately test emotion or valence specific effects of vasopressin modulation and SRX246, as it is possible that real effects on the BOLD response to other emotion conditions were not detected.

3.4*: Secondary Analyses: Exploratory analyses were conducted regarding the main effects of SRX246 on regions of interest identified in 3.4, for emotion conditions other than Angry. These included happy, fear, and neutral faces vs. fixation cross, in addition to an all faces condition and an aversive faces (anger + fear) condition. To manage Type II error given the power limitations of the study design, exploratory analyses were restricted to extracted data from anatomical regions identified in **Section 3.4.** Results of ANCOVA, comparing IN vasopressin versus IN placebo for Session 2, covarying for Session 1, in the five ROIs for each emotion condition are provided in **Table 4** below:

	Right Putamen	Left Putamen	Right Precuneus	Right Anterior Cingulate	Right TPJ
Fear vs. Fixation	F (1,26) = .001<br>p = .95	F (1, 26) = .210<br>p = .65	F (1, 26) = 1.3<br>p = .27	F (1, 26) = .510<br>P = .48	F (1, 26) = .643<br>p = .43
Happy vs. Fixation	F (1, 26) = .086<br>p = .77	F (1, 26) = .028<br>p = .87	F (1, 26) = .399<br>p = .53	F (1, 26) = .242<br>P = .63	F (1, 26) = .358<br>p = .56
Neutral vs. Fixation	F (1, 26) = .072<br>p = .79	F (1, 26) = .443<br>p = .51	F (1, 26) = .007<br>p = .935	F (1, 26) = .814<br>p = .375	F (1, 26) = .023<br>p = .88
All Faces vs. Fixation	F (1, 26) = 1.475<br>p = .236	F (1, 26) = 1.92<br>p = .54	F (1, 26) = .049<br>p = .83	F (1, 26) = 2.160<br>p = .154	F (1, 26) = .998<br>p = .33
Anger +Fear vs. Fixation	F (1, 26) = 4.159<br>p = .05	F (1, 26) = .308<br>p = .58	F (1, 26) = .454<br>p = .51	F (1, 26) = 8.902<br>p = .01	F (1, 26) = 4.160<br>p = .05

Table 4: Results of ANCOVA for Session 2 Average BOLD Parameter Estimates Covarying for Session 1 in Regions of Interest: Exploratory analyses were conducted for emotion contrasts other than Angry vs. Fixation, including an average of all emotion conditions (All Faces) and aversive emotions (Anger + Fear). All tests are 2-tailed, uncorrected. Significant effects are found for the Anger + Fear vs. Fixation condition in the right putamen, right anterior cingulate, and right temporoparietal junction.

Summary: The results of exploratory analyses suggest that the main effects of SRX246 on tested ROIs are strongest during processing of facial expressions of anger. However, significant effects (uncorrected for multiple measures) were also detected in the right putamen, right anterior cingulate, and right TPJ during processing of aversive faces. It is possible that the effects of SRX246 on the brain are seen during processing of aversive facial emotions, rather than angry faces specifically. Given the limited power of the study, it is possible that effects of SRX246 on brain processing of other facial emotions were not detected.
